# Spatial Representation of the Workspace in Blind, Low Vision, and Sighted Human Participants

**DOI:** 10.1177/2041669518781877

**Published:** 2018-06-17

**Authors:** Jacob S. Nelson, Irene A. Kuling, Monica Gori, Albert Postma, Eli Brenner, Jeroen B. J. Smeets

**Affiliations:** Department of Human Movement Sciences, Vrije Universiteit Amsterdam, the Netherlands; U-VIP Unit for Visually Impaired People, Istituto Italiano di Tecnologia, Genoa, Italy; Department of Experimental Psychology, Helmholtz Institute, Utrecht University, the Netherlands; Department of Human Movement Sciences, Vrije Universiteit Amsterdam, the Netherlands

**Keywords:** blindness, low vision, length reproduction, coordinate system, haptics, proprioception

## Abstract

It has been proposed that haptic spatial perception depends on one’s visual abilities. We tested spatial perception in the workspace using a combination of haptic matching and line drawing tasks. There were 132 participants with varying degrees of visual ability ranging from congenitally blind to normally sighted. Each participant was blindfolded and asked to match a haptic target position felt under a table with their nondominant hand using a pen in their dominant hand. Once the pen was in position on the tabletop, they had to draw a line of equal length to a previously felt reference object by moving the pen laterally. We used targets at three different locations to evaluate whether different starting positions relative to the body give rise to different matching errors, drawn line lengths, or drawn line angles. We found no influence of visual ability on matching error, drawn line length, or line angle, but we found that early-blind participants are slightly less consistent in their matching errors across space. We conclude that the elementary haptic abilities tested in these tasks do not depend on visual experience.

## Introduction

The environment is filled with rich, multisensory stimuli. In humans, the visual system plays a crucial role in information processing, especially at distances that cannot be physically reached. For nearby stimuli, touch and proprioception help to form a complete picture of the world. Visual information plays an important role in the development of various spatial abilities, as it provides information about the position and arrangement of the surrounding environment that the nervous system would not otherwise necessarily have access to.

### Effects of Early Blindness

Several studies suggest that the brain may respond to visual deprivation by improving the sensitivity of the remaining sensory systems. Early-blind individuals show enhanced skills for some auditory tasks such as localization of a single sound on the horizontal plane (Lessard, Paré, Lepore, & Lassonde, 1998; Röder et al., 1999; Voss et al., 2004). Early-blind individuals have also been shown to have lower spatial thresholds for tactile discrimination of stimuli (Brown & Stratton, 1925; Jones, 1972), contrary to an earlier report (Seashore & Ling, 1918). Postma, Zuidhoek, Noordzij, and Kappers (2007) found that early-blind and late-blind adults were significantly faster than blindfolded-sighted adults at placing wooden shapes into a board with corresponding cutouts, as well as in placing the same shapes in the correct positions on a flat surface from memory. However, some studies did not find superior performance in haptics due to absence of vision. Jones and Vierck (1973) did not find a difference in threshold between blind and sighted individuals in detecting which of two stimuli pressed against their arm was longer. Gori et al. (2010) found that visually impaired children (one late-blind and the rest early-blind) were slightly better than sighted children at tactile object size discrimination but that they were drastically worse at object orientation discrimination, perhaps suggesting that orientation perception relies more heavily on the visual system.

On the other hand, blind individuals unsurprisingly suffer from deficits in a number of skills ranging from auditory and spatial localization to navigation (see Cuturi, Aggius-Vella, Campus, Parmiggiani, & Gori, 2016 for a review on this topic). Recent studies show that early-blind individuals fail in localizing sounds under particular auditory settings (Gori, Sandini, Martinoli, & Burr, 2013; Vercillo, Milne, Gori, & Goodale, 2015). Hollins and Kelly (1988) observed an interesting discrepancy between early-blind adults’ ability to point to an object on a table after having walked partway around the table and their ability to place the object in its original position after having walked around the table; whereas blindfolded-sighted people performed well in both of these tasks, early-blind adults performed well at replacement but not at pointing. It has also been shown that while early-blind and blindfolded-sighted adults are both better at remembering symmetrical configurations of blocks on a table than at remembering asymmetrical ones, the early-blind group was worse at remembering vertically symmetrical configurations than horizontal ones (Cattaneo et al., 2010). The authors proposed that the blindfolded-sighted adults were able to benefit from enhanced attention to vertical symmetry granted by the visual system.

In some cases, it does not appear to matter when a person first became blind to have reduced performance at a skill. Gentaz and Hatwell (1998) observed no differences between early-blind and late-blind adults in their ability to match the orientation of a rod to that of a reference rod but concluded that their reliance on gravitational cues for information differed from that of sighted participants (Gentaz & Hatwell, 1996).

### Haptic Space Perception

When describing locations of objects, people can rely on two kinds of frames of reference. Using an egocentric reference frame, one might describe objects as “one meter to my left” or as “ten degrees to my right.” Using an allocentric reference frame, one relies on external references, such as “one meter to the left of the laptop” or “below the table.” To manipulate an object, one needs to know its egocentric location, but when remembering or describing its position, it can be more useful to rely on the allocentric location because it does not depend on one’s own spatial orientation. There is evidence that eye-centered coordinates are fundamental for the association of sensory signals (Cohen & Andersen, 2002; Jay & Sparks, 1987; Pouget, Deneve, & Duhamel, 2002), and the visual modality might offer a spatial background for remapping sensory information to obtain stable externally defined coordinates when one’s spatial orientation (including that of the eyes) changes. When the visual signal is missing, such spatial remapping may not occur. In agreement with this idea, congenitally blind individuals are not subject to the “crossed hand illusion” (Röder, Rösler, & Spence, 2004), presumably because they do not have the perceptual conflict with an externally anchored reference system for tactile stimuli. There is also experimental evidence that congenitally blind individuals do not remap auditory stimuli onto externally defined coordinates (Röder, Kusmierek, Spence, & Schicke, 2007). It has been suggested that it is specifically *early-blind* people whose spatial experience of the world depends largely on egocentric reference frames rather than allocentric reference frames (Iachini, Ruggiero, & Rutolo, 2014; Pasqualotto, Spiller, Jansari, & Proulx, 2013). Therefore, one might generally expect the early-blind to rely more on egocentric reference frames for judging spatial relations.

It has been shown that two bars on a tabletop that are felt to be parallel can actually have dramatically different angles from one another and that this difference scales almost linearly with the angular distance between them (Kappers, 2003; Kappers & Koenderink, 1999). The researchers concluded that participants’ perception of haptic space relied on a combination of allocentric and egocentric reference frames, the latter of which was centered on the hand. We expect that similar deviations will be found for drawing lines in a specified direction.

Using a similar parallel-bars paradigm, Postma, Zuidhoek, Noordzij, and Kappers (2008) showed that when instructed to wait 10 s between exploring a reference bar and rotating a test bar, early-blind participants relied more on an egocentric reference frame than late-blind and blindfolded-sighted adults. Based on this increased reliance on an egocentric reference frame, we expect early-blind adults who are instructed to draw straight frontoparallel lines starting at different distances from the body midline to draw lines with more strongly deviating angles than sighted people. Similarly, when instructed to draw lines of a given length at different distances from the body, early-blind adults’ drawn lines may be longer at larger distances from the body due to relying more heavily on an egocentric reference frame, at least if such a reference frame is in polar coordinates (direction and distance).

Kuling, Brenner, and Smeets (2016) have shown that sighted adults are not always accurate at placing their hand on a table above the location of their other, unseen hand under the table. Kuling, van der Graaff, Brenner, and Smeets (2014) showed that matching a target using a handheld pointing tool does not result in differences in magnitude or variability of error compared with using one’s own fingertip in a visuoproprioceptive matching task. Furthermore, while it has been shown that using proprioception alone in a matching task is less effective than combining sight and proprioception (Van Beers, Sittig, & Denier van der Gon, 1999), it also remains to be tested whether early-blind adults’ proprioceptive accuracy and precision in a position matching task are worse than that of blindfolded-sighted adults. We speculate that this matching error is already ameliorated by way of the visual system and that early-blind adults may show greater matching errors than blindfolded-sighted adults due to a lack of visual experience.

## Methods

### Participants

All participants in the experiment (*n* = 132, mean age 48.5 years, *SD* = 17.0, 22 left-handed, 86 females) were attendees of the “ZieZo Beurs,” a convention for blind and visually impaired people, who visited the Utrecht University booth that was set up for the event. All participants were naïve to the purpose of the experiment, with the exception of one participant with normal vision who was also an author. Participants were given an explanation of the task and were asked for their informed consent. Once verbal consent was given, they were asked several general questions about their vision, age, and handedness.

For the analyses, the participants were grouped based on level of vision. The five groups were referred to as early-blind, late-blind, low vision, high vision, and sighted. Early-blind participants were all born completely blind and have never experienced any level of vision. Late-blind participants all became completely blind either due to a congenital condition leading to gradual loss of vision or due to an accident. The age of total vision loss in the late-blind group ranged from 2 to 47 years. Participants in the low vision group reported having some degree of visual perception, but no more than 10% in either eye. Participants in the high vision group reported having more than 10%, but no more than 80% vision in either eye. Participants in the sighted group reported having normal or corrected-to-normal vision. Level of vision was determined by self-report. The low vision and high vision groups consisted of participants who possessed congenital visual deficits as well as deficits acquired later in life by various means.

Due to the nature of the convention, participants were not actively matched between groups, and it was not possible to perform an in-depth intake session with each participant. We therefore lacked information regarding our participants’ specific visual capacities, as well as haptic processing abilities such as the ability to read braille, which could potentially have an impact on their ability to perform the tasks described here. Details of the participant groups can be found in Table 1.

**Table 1.** Group-Level Characteristics of Participants.

Group	*n*	Age (*M* ± *SD*)	Left-handed	Female
Early-blind	7	43 ± 17	1	5
Late-blind	9	64 ± 11	2	5
Low vision	30	51 ± 12	8	15
High vision	31	55 ± 17	6	18
Sighted	55	42 ± 18	5	43
Total	132	49 ± 17	22	86

### Experimental Setup and Procedure

**Figure 1.**
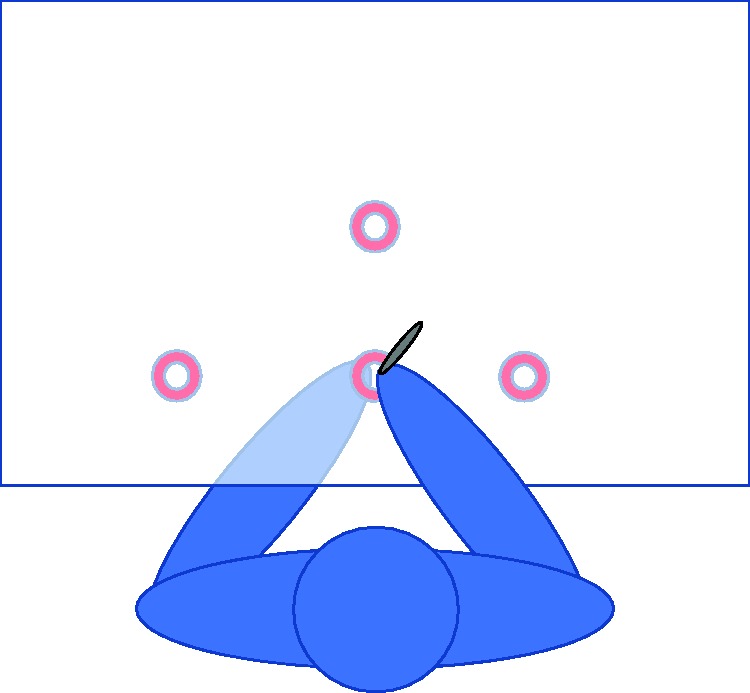
Top view of a participant at the experimental table. The haptic targets, placed under the table, are indicated by red rings, contacted by the index finger of the nondominant hand. The dominant hand was holding a pen above the table. Target size is misrepresented here for the sake of visibility.

The setup consisted of a 100 × 65 cm wooden board on which flip chart paper sheets could be mounted. The board rested on two supporting trestles, resulting in a table (Figure 1). Four metal washers were placed under the board as haptic targets for the nondominant hand. The washers had a diameter of 3 cm with a 1-cm gap in the middle such that participants could comfortably guide their fingertip to the center of the washer. Two of these washers were placed along the center of the board, at 15 and 35 cm away from the long edge (henceforth referred to as the near and far targets, respectively). The remaining two washers were placed 20 cm to the right and 26.6 cm to the left of the near target, respectively (henceforth referred to as the side targets). The different lateral distances of the two side targets were an error that we compensated for in the analysis. The right-side target was used only by right-handed participants, and the left-side target was used only by left-handed participants. An aluminum bar of 10 × 1 × 1 cm served as a reference object, which participants held briefly at the beginning of the experiment.

Participants sat in a chair in front of the setup and were blindfolded. The participants sat such that their midsagittal body plane was aligned with the near and far haptic targets. The task was explained, and participants were invited to explore the table with their hands to get a feel for the positions of the haptic targets. Participants were asked to confirm that they could reach all three targets before continuing. The experimenter handed the reference object to the participants and instructed them to get a feel for the length of the object. Participants were allowed to hold it for several seconds in their dominant hand and manipulate it. They were not allowed to set it down on the table, run their finger along the length of it, or touch it with their nondominant hand. Most participants held the object between their thumb and pinkie finger to get a feel for the object’s length; they were not restricted to holding it in in their palm. The experimenter explained that the participants’ task would be to draw lines of this length. The experimenter took the reference object back once the participants said they had an idea of the length (typically after 5 to 10 s) and confirmed that they understood the task.

The experimenter then placed an ink marker in the participants’ dominant hand and guided the index finger of their nondominant hand toward one of the three haptic targets. Participants were asked to use the marker to indicate on the paper where their nondominant hand was placed, and from this point draw a line with the same length as the reference object. Three targets were used: the near and far targets, as well as the side target that corresponded with their dominant hand. Right-handed participants were asked to draw all lines from left to right, and left-handed participants were asked to draw all lines from right to left. All participants thus drew three such lines, one from each of the three targets in a randomized order, without any practice or familiarization. Because we did not know in advance how many participants we would be able to recruit, we did not counterbalance the order of the targets across participants. After a participant had finished the task, the sheet of paper was removed, and a fresh sheet was placed on top of the setup for the next participant.

### Analysis

For each participant and each target, we determined the position of the dot at the start of the line, the matching error (i.e., the two-dimensional vector between the haptic target and the dot at the start of the line), the length of the drawn line (i.e., the shortest distance between the start and the end of the drawn line), and the angle of the drawn line relative to the coronal plane. For each of our analyses, we examined whether the early-blind performed differently than the sighted group. We used one-tailed independent samples *t* tests to do so, as we expected early-blind participants to show greater matching errors, length ratios between near and far target lines, and angular differences between near and side target lines than sighted participants. In cases where a significant difference was found, we also performed one-tailed *t* tests to determine whether the early-blind group differed from the other groups.

In this article, four questions are investigated:

1. Do early-blind participants show greater matching errors than sighted participants?
2. Do early-blind participants show less matching error consistency than sighted participants?
3. Do participants draw longer lines from far targets than from near targets, and is this difference larger for early-blind than for sighted participants?
4. Do participants draw differently oriented lines from the near and side targets, and is the angular difference larger for early-blind participants than for sighted participants?

#### Matching errors

We first analyzed the magnitude of the matching errors of each participant group, ignoring the direction of the error. To determine whether participants’ matching errors (both magnitude and direction) were consistent across targets, we calculated a consistency value as described in previous studies (Kuling et al., 2016; Kuling, van der Graaff, Brenner, & Smeets, 2017). The consistency value was determined for all three combinations of the three target positions (i.e., ME_near_ compared with ME_far_, ME_far_ compared with ME_side_, and ME_side_ compared with ME_near_), after which an average value for each participant was calculated. To determine a meaningful baseline, we also calculated a chance consistency error by comparing every individual matching error with every other matching error; a value above this baseline indicates that participants are at least somewhat consistent in their errors across targets (Kuling et al., 2016, 2017). We analyzed whether early-blind participants had significantly larger matching error magnitudes and lower consistency values than sighted participants.

#### Line analysis

For the comparison of the drawn lines, we looked at drawn line lengths starting from the near and far targets, as well as drawn line angles starting from the near and side targets. For the lengths, we analyzed whether the ratio of the far target line length to the near target line length was larger than 1, and whether it was larger for the early-blind than for the sighted participants. For the angle, we examined whether the angular differences between drawn lines at the near and side targets were greater for early-blind participants than for sighted participants. To correct for the difference in relative position of the left- and right-side targets, we multiplied left-handed participants’ values by 20 cm/26.6 cm, as it is known that the angle at which a line is felt as frontoparallel scales linearly with distance from the body midline (Kappers, 1999).

## Results

The data of three participants (two low vision and one sighted) were completely excluded from the analysis because they drew lines from arbitrary starting points and reported that they did not realize they needed to match the haptic targets or pay attention to the reference object’s length. Data from a further 10 participants (three late-blind, two low vision, three high vision, and two sighted) were excluded from analyses of the drawn lines, but not from that of the matching task, due to the participants drawing one or more lines in the wrong direction or, in two cases, starting their drawn lines from somewhere other than the (correctly located) haptic targets.

**Figure 2.**
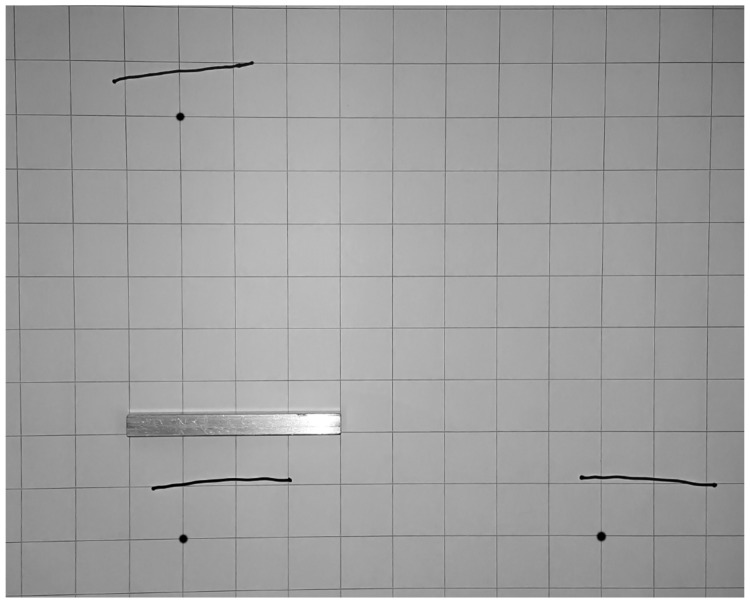
Example experiment sheet from a 20-year-old right-handed male in the sighted group with representative performance. Black dots indicate the location of the haptic targets under the table, and the reference object is displayed above the near target for comparison. The target dots and reference object were not present on the paper during the experiment. The grid squares have 2.5 cm sides.

The raw data of an example representative participant (Figure 2) show matching errors that are quite consistent over the three targets: The drawn lines all started about 3 cm from the target position (farther away and a bit to the left). Furthermore, the lengths of this participant’s drawn lines are about 6 cm, considerably shorter than that of the 10-cm reference object. The line drawn from the side target is rotated a few degrees clockwise relative to the one drawn from the near target.

**Figure 3.**
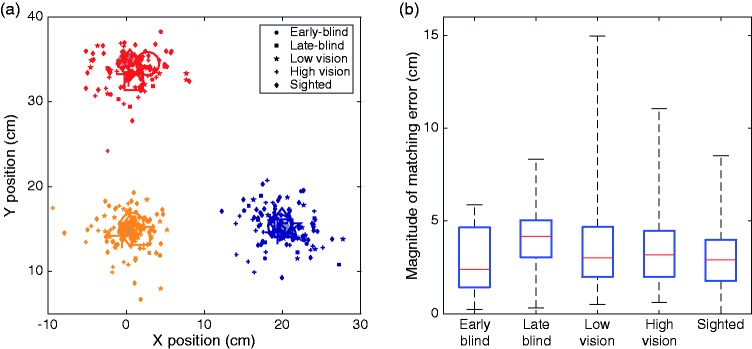
Matching errors. (a) Matching errors of all right-handed participants. Left-handed participants’ data are not shown here due to the positioning of the left-side target. Target positions are denoted by large gray disks at (0,15); (0,35); and (20,15) cm. The large colored symbols denote mean matching errors for each group. (b) Magnitudes of matching error for all participants. Box plots show median (red line), interquartile range (blue box), and full range (black marks).

The individual matching errors for right-handed participants show quite some variability, but there was no evident difference between groups (Figure 3(a)). We focus our analysis on the magnitudes of the matching errors (Figure 3(b)). We found that early-blind participants did not have greater mismatch magnitudes than sighted participants (one-tailed independent samples *t* test), *t*(59) = −0.184; *p* = .573.

**Figure 4.**
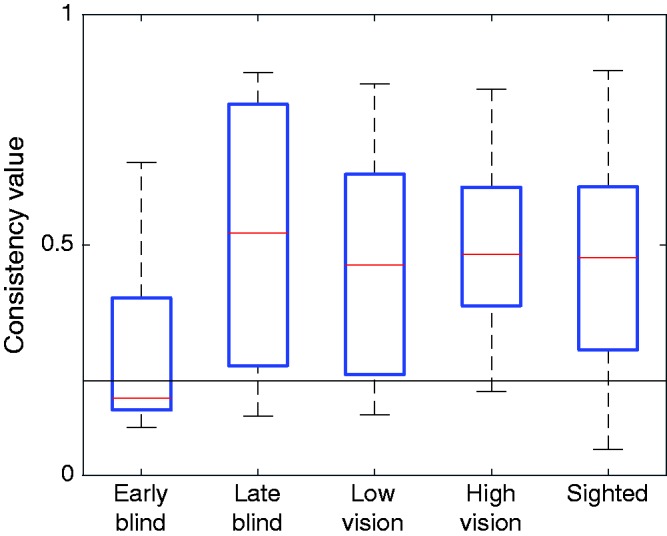
Matching error consistency values for all participants. The horizontal line indicates the median chance consistency value, 0.207. For an explanation of the calculated consistency values, see Kuling et al. (2016, 2017).

The consistency of the early-blind participants’ errors across target positions is close to chance level, whereas the other groups show more consistency (Figure 4). Early-blind participants showed a significantly lower consistency value than sighted participants (one-tailed independent samples *t* test), *t*(59) = 2.264; *p* = .013. The early-blind also showed a significantly lower consistency value than the other three groups—late-blind: *t*(14) = 1.189, *p* = .040; low vision: *t*(33) = 1.183, *p* = .038; high vision: *t*(36) = 2.894, *p* = .003.

**Figure 5.**
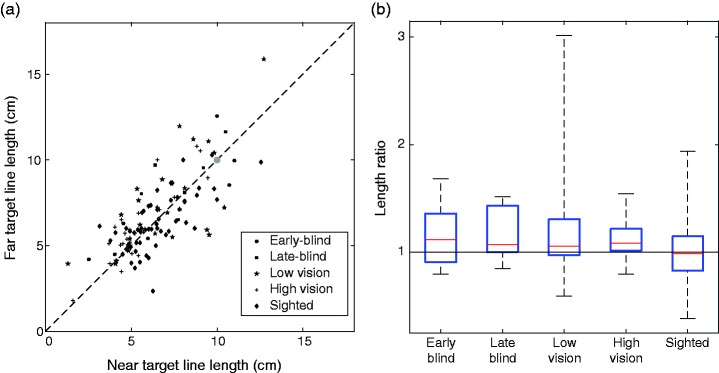
Length reproduction. (a) Comparison of drawn line lengths from the near and far targets for all participants. The dashed line indicates unity, and the gray dot indicates perfect reproduction of line length from both near and far targets (10 cm). (b) Box plot of length ratios between the lines drawn from the far and near targets.

Most participants drew lines that were considerably shorter than the 10 cm length of the felt object (Figure 5(a)): on average only 6.59 cm (66% of the reference object’s length). They drew lines of similar lengths at the different distances: The average ratio of the far and near line lengths was 1.08 (Figure 5(b)). Early-blind participants’ line length ratios were not significantly larger than those of sighted participants (one-tailed independent samples *t* test), *t*(57) = 1.50, *p* = .07. Furthermore, neither early-blind nor sighted participants’ line length ratios were significantly larger than 1.0 (one-tailed one sample *t* test)—early-blind: *t*(6) = 1.25, *p* = .13; sighted: *t*(51) = −0.04, *p* = .52—indicating that there was no tendency to draw lines of a certain angular length, rather than ones that match the reference object’s actual length.

**Figure 6.**
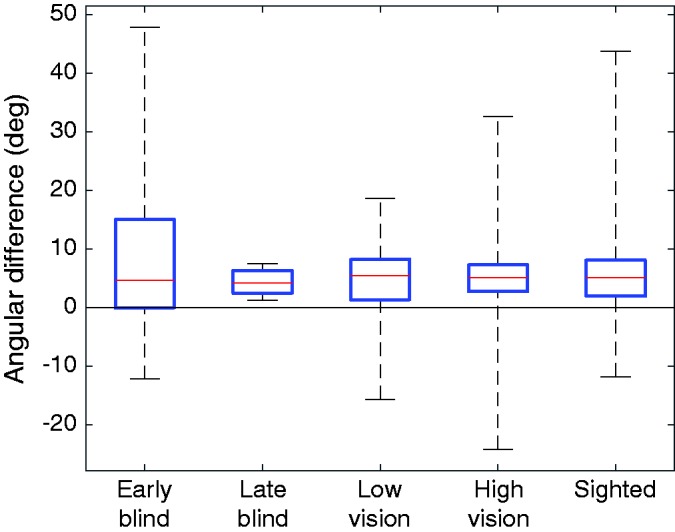
Difference in angle between near and side target lines for all participants. A positive value indicates a more clockwise orientation for a more rightward target position. Left-handed participant data have been normalized to correspond to a 20-cm difference between the near and side targets.

Lines from the side target were drawn in a systematically different direction than those from the near target (Figure 6). The 5.3° angular difference was significant across groups (clockwise for right-handed participants and counter-clockwise for left-handed participants; one-tailed paired-samples *t* test), *t*(118) = 6.26, *p* < .001. The angular difference between lines drawn from the near and side targets was not significantly larger for early-blind than for sighted participants (one-tailed independent samples *t* test), *t*(57) = −1.00, *p* = .84. These findings indicate that while most participants draw lines at different angles when beginning in different directions from their body, in accordance with them relying to some extent on a (polar) egocentric representation, the angle was not particularly large in early-blind participants, suggesting that the early-blind do not rely more strongly on such a representation.

## Discussion

We did not observe the differences that we anticipated between early-blind and blindfolded-sighted participants. Both early-blind and sighted participants drew lines that are much shorter than the felt bar and were drawn at angles that similarly depended on eccentricity. As these effects were similar across groups, they can better be attributed to a general property of haptic perception than to an effect that depends on visual experience. The sizes of our participant groups were relatively small, so we may have failed to detect small but real effects. Our interest was in clear-cut effects of lack of visual experience, for which we investigated differences between early-blind and sighted participants. The expected systematic effects of lack of visual experience were not evident in our sample.

We are not aware of a theory that makes specific predictions for how the performance in our task would depend on details of the impairment such as the age of onset of blindness. Given the time constraints inherent to data collection at a convention, we did not collect data on the precise history of our participants’ visual impairments. Therefore, we cannot perform any exploratory analysis on this. Similarly, we did not intend to investigate biases caused by other factors than being blind, so we did not examine whether participant height, hand size, or arm length played a role in task performance.

### Matching Errors

We observed average matching errors of 3 cm, which is in line with previous reports (Haggard, Newman, Blundell, & Andrew, 2000; Kuling et al., 2016; Von Hofsten & Rösblad, 1988). What is particularly interesting about this is that participants who have never experienced vision are just as accurate at matching their hand positions as blindfolded-sighted participants, indicating that vision does not play a role, even indirectly, in this task. The low consistency value in early-blind participants (Figure 4) is remarkable. While the actual size of the matching error is well in line with that of other groups (Figure 3(b)), this error was not consistent across target positions in the early-blind participants, in contrast to the other groups. It is possible that growing up with visual input plays a role in developing this consistency, which early-blind people would therefore lack. However, given that the observed range of consistency values among early-blind participants falls within that of nearly all other groups, and given that the early-blind group consisted of only seven participants, it is not clear whether this difference is indicative of an actual matching deficit in early-blind individuals, or is simply due to chance.

### Length Reproduction

It is worth pointing out that our participants fall well short of the desired length when drawing lines, regardless of level of visual experience or starting position of the line (Figure 5(a)). This appears to be in contrast with the findings of Hermelin and O’Connor (1975), who observed that when congenitally blind and blindfolded-sighted children were asked to reproduce vertical movements traveled by their hand, they were accurately able to do this for path lengths of 10 to 30 cm. Their task relied on kinesthetic movement information rather than on static tactile information. Our task involves the transformation of static tactile information (a felt bar in one’s hand) to dynamic proprioceptive information. It is possible that this transformation is responsible for the systematic undershoot. They noticed a difference in performance between blind and sighted participants when reproducing the longer movement distances (25–30 cm): The blind participants undershot these more than the sighted. As a hand cannot statically feel a length of 25 to 30 cm, this difference for longer movement distances cannot be tested with our paradigm of a handheld length.

Previous work has shown that the horizontal–vertical illusion exists in the haptic domain as well as the visual domain. Heller and Joyner (1993) demonstrated that early-blind adults show evidence for a vertical compression of haptic space, but the size of the effect was small compared with the undershoot that we observe in our data. As such, we do not expect that the lengths of our participants’ drawn lines would be substantially longer if we altered the orientation in which they were instructed to hold the reference object.

During the review process, it was suggested that hand size may have influenced participants’ perception (a smaller hand might lead to the percept of a longer bar) and thus on the length of the drawn lines. We did not measure hand size during the experiment, but analyzed our data in light of gender (on average women have smaller hands). Indeed, it appears that women drew somewhat longer lines than men: The median line length (calculated over all groups) was 6.4 cm for women and 5.8 cm for men.

Lederman, Klatzky, and Barber (1985) tested participants’ ability to reproduce a distance between two points by moving their index finger after traveling along an indirect path between the two points with that finger. They observed that early-blind participants tended to make larger reproductions of the distance than late-blind participants. They also observed an overshoot in reproduced length for all participants for lengths up to 25 cm and an undershoot for greater distances. Because Lederman et al.’s participants traced a path with their finger while attending to the distance between the starting and ending positions, whereas our participants acquired length information without any tracing motion, our participants’ systematic undershoot of the target distance cannot be related to this overshoot.

Moscatelli, Naceri, and Ernst (2014) tested whether participants could accurately reproduce lengths of a triangle path felt by a tactile stimulation device. Their participants could much more accurately reproduce the perceived displacement than our participants reproduced perceived length: Their reported 98% accuracy is far higher than our 66% accuracy. A reason for this difference could be that the two experiments differed in how participants perceived the distances they had to reproduce. Their participants rested their index finger on a spherical device that rotated while the finger remained stationary on top of it, providing the tactile experience of moving a finger over a surface. Our participants were allowed to manipulate a three-dimensional reference object in their dominant hand, but they were not permitted to run a finger along the length of the object.

The findings of Moscatelli et al*.* (2014), Lederman et al. (1985), and Hermelin and O’Connor (1975) all show that people are able to reproduce a given length reasonably accurately. In all these experiments, the participants could judge the length based on haptic motion information. As our participants made systematic errors and lacked this information, it may be necessary to have haptic motion information to accurately reproduce lengths in a drawing task. Irrespective of the reason for the distances being underestimated in our study, the fact that we observed no differences between early-blind and sighted participants in the ratio of lengths of lines drawn from the near and far targets (Figure 5(b)) suggests that for this task, the early-blind do not rely more on an egocentric coordinate system than the sighted.

### Parallel Reproduction

Kappers (2004) demonstrated that haptic space perception relies on a combination of egocentric and allocentric reference frames, explaining the tendency of the angle of the test bar to lie somewhere between what would be considered parallel in purely allocentric or purely egocentric reference frames. Her experiments to map out haptic space were far more extensive than ours, but the differences that we see in drawn line orientation (Figure 6) are of similar magnitude to what she observes. Van Mier (2013) tested sighted adults’ ability to draw a line parallel to a felt but unseen bar. Despite the fact that her participants were allowed to see the line they were drawing, there was still an angular difference between the orientation of the reference bar and that of the drawn line. Although this difference is smaller than what she observed in her purely haptic parallelity task, it still suggests that the reference frame experienced by each hand is important. This similarity is particularly interesting because we never explicitly told our participants to draw parallel lines; we simply always instructed them to draw the lines “from left to right” (or from right to left, if the participant was left-handed).

We observed no differences between early-blind and sighted participants in angular differences between the drawn lines from the near and side targets. This confirms that for this task, the early-blind do not rely more on an egocentric coordinate system than the sighted. The small but significant difference in angle between the drawn lines from the near and side targets can therefore be said not to rely on visual experience. Previous work has shown that women make substantially larger errors than men when matching the orientations of two haptically felt bars (Kappers, 2003). We did not observe a similar effect in the orientation of the drawn lines. Our female participants even showed a slightly smaller median angular difference than our male participants (5.0° and 5.5°, respectively).

Postma et al. (2008) observed differences between early-blind, late-blind, and sighted participants in a delayed bar parallelity task. When asked to wait 10 s between feeling a reference bar and setting a test bar parallel to it, blindfolded-sighted participants performed better than late-blind participants, who in turn performed better than early-blind participants. The fact that we do not observe a similar effect, despite a delay between feeling the reference object and drawing the lines, likely stems from the fact that we do not explicitly ask our participants to consider the egocentric relation of the object to the participant. Instead, we only asked them to consider the length of the object, regardless of orientation, so the angle of the participants’ drawn lines should have no relation to the angle at which they felt the object.

## Conclusion

We expected that early-blind participants would rely more on information processed within an egocentric reference frame but found no indication of this in our results.

All data pertaining to this study, both analog and digital, are freely available for review. Interested parties are invited to contact the first author for specific questions or access to the data and materials.
